# Can hyperleukocytosis be caused by a non-hematologic condition? A 10-year retrospective tertiary-care center cohort study

**DOI:** 10.1080/07853890.2026.2636347

**Published:** 2026-02-28

**Authors:** Elisavet Moutzouri, Emmanuel Häfliger, Katarzyna Aleksandra Jalowiec, Ioannis Chanias, Martin Andres, Annatina Schnegg-Kaufmann, Adrian De Angelis, Marie-Noëlle Kronig, Thomas Pabst, Ulrike Bacher, Natalia Baran, Anne Angelillo-Scherrer, Sara Christina Meyer, Michael Daskalakis, Alicia Rovó

**Affiliations:** ^a^ Department of Hematology and Central Hematology Laboratory, Inselspital, Bern University Hospital, University of Bern, Bern, Switzerland; ^b^ Department of Oncology, Inselspital, Bern University Hospital, University of Bern, Bern, Switzerland; ^c^ Department of Internal Medicine, Sanford School of Medicine, University of South Dakota, Sioux Falls, South Dakota, USA

**Keywords:** Hyperleukocytosis, hematologic malignancies, mortality, non-hematologic causes

## Abstract

**Background:**

Hyperleukocytosis (HL) is defined as a white blood cell counts typically exceeding 100 G/L and is commonly associated with hematologic malignancies. Efforts to identify and characterize non-hematologic causes of HL have been limited and insufficiently addressed in the literature.

**Methods:**

We conducted a retrospective analysis of adult patient records from 2013 to 2023, screened for cases of HL with a primary focus on the characterization of non-hematologic causes.

**Results:**

Out of 878,967 adult patients, 375 patients had HL; 90 were excluded due to opting out of general consent, leaving 285 (median age 66.8 years; 32% female) for analysis. Of these, 276 (97%) had an underlying hematologic malignancy, most frequently chronic lymphocytic leukemia (35.4%), followed by other B-cell lymphomas, acute myeloid leukemia, and chronic myeloid leukemia. Non-hematologic causes accounted for the remaining 3% (*n* = 9), all associated with advanced solid tumors and predominantly paraneoplastic in nature. Isolated infection-related HL was not observed in this cohort.

**Conclusions:**

The presence of HL should immediately raise the possibility of a hematologic disease, though a small subset stems from non-hematologic causes. Consequently, HL should prompt immediate peripheral blood smear review and early hematology consultation. Isolated infection-related HL was not observed in this cohort and should be interpreted as a cohort-specific observation.

## Introduction

Leukocytosis refers to an increased white blood cell (WBC) count of more than 11 G/L. Leukocytosis can result from various primary or secondary causes [1–3]. While primary causes are related to hematologic conditions, secondary causes are described in association with infections, cytokine-secreting solid tumors, iatrogenic G-CSF-related, steroid demargination, severe stress states and as physiological finding observed postsplenectomy [4,5]. There is no internationally accepted consensus for the numerical definition of moderate or severe leukocytosis. Thus, some publications consider moderate leukocytosis as a threshold of 25–40 G/L, while values above 40 G/L are described as severe leukocytosis. In this range, the most common etiology is infections, frequently in severely ill patients (pneumonia, pyelonephritis, and abscesses). Perhaps special mention should be made of what is described as a leukemoid reaction characterized by a transient increase in WBCs, usually with neutrophilia and a left shift of the myeloid series, which can mimic leukemia; however, unlike leukemia, blasts are absent. Leukocytosis >100 × G/L is in general referred to as hyperleukocytosis (HL). However, the definition of hyperleukocytosis (HL) is used differently depending on the specific hematologic diseases. For example, in patients with promyelocytic leukemia, HL is considered to be a WBC count above 50 G/L [6], while reporting of HL in lymphoma cases is commonly associated with extremely high WBC levels, sometimes above 400 G/L [7,8]. These disparities are explained by pathophysiological factors and the potential for leukostasis: While immature myeloid cells and monocytes have a high risk of causing leukostasis, small lymphocytes can reach much higher counts without producing clinical symptoms.

The primary objective of this study was to estimate the proportion of non-hematologic causes among adults with HL, with secondary objectives including clinical characterization and short-term outcomes.

## Methods

We performed a retrospective study searching the database of patients’ medical records (Ipdos), at a tertiary university hospital. In line with the Swiss Human Research Act (HFG) and the decision of the local ethics committee in Bern (KEK Number 2023-01984), all patients included either provided general consent or did not exercise their right to opt out. University hospital of Bern is one of the largest hospitals in Switzerland, providing care for over 500,000 patients annually and serving as a major academic center. We performed a stepwise search, starting with the identification of patients with HL by analyzing laboratory values of all adult patients seen in the hospital between 2013 and 2023. Patients with at least one episode of HL and no written objection to the general consent of the hospital were further evaluated. If a patient had multiple episodes of HL, their highest count was considered for the analysis. Records from both inpatients and outpatients were analyzed. Only validated laboratory results reported by the central hospital laboratory were included. At our institution, routine clinical practice requires confirmatory repeat testing or manual verification for extreme WBC values; therefore, isolated analytical artifacts are considered unlikely. Hemoconcentration was not used as an exclusion criterion but was assessed during individual chart reviews when clinically relevant. Differential counts (manual or automated) at the time of peak WBC when available - or at the closest available date -were extracted. Peripheral blood smear reviews and blast flagging were performed according to routine clinical care; no additional retrospective smear adjudication was undertaken. HL cases were initially identified through electronic laboratory queries and subsequently classified using predefined ICD-10 codes. Following HL identification, all diagnoses recorded for each patient were extracted. Hematologic malignancies were identified using ICD-10 codes C81-C96 (malignant neoplasms of lymphoid, hematopoietic, and related tissue) and D47 (other neoplasms of uncertain or unknown behavior of lymphoid, hematopoietic, and related tissue), including all subcodes. Patients without any of these codes were provisionally categorized as non-hematologic HL and subsequently underwent detailed individual chart reviews by two authors. This review included clinical notes, oncologic history, imaging findings, microbiological results, medication exposures, including granulocyte colony-stimulating factor (G-CSF), laboratory trends, and overall clinical course.

When multiple potential contributors (e.g. infection, solid tumor, recent G-CSF exposure) were present, attribution of the primary cause was based on the temporal relationship to HL onset and the treating physician’s documented clinical assessment. Where multiple etiologies coexisted, the most likely primary cause was adjudicated by chart reviews using predefined clinical criteria, including temporality, treatment exposure, and physician documentation. For example, leukocytosis present prior to G-CSF administration was not attributed to G-CSF, whereas leukocytosis occurring shortly after G-CSF exposure was considered treatment-related. Likewise, temporal alignment with clinical and microbiological evidence was used to attribute leukocytosis to infection. Isolated infection was defined as microbiologically or clinically documented infection in the absence of advanced malignancy or recent leukocyte-stimulating therapy. Laboratory parameters such as CRP and LDH were reported when available. If not available at the time of peak HL, last value availability preceding the HL peak was reported. Missing values were explicitly indicated, and no imputation was performed due to the descriptive nature of the study. Data on early mortality, defined as 30-days all-cause mortality following HL peak were also extracted from the medical records. There was no formal adjudication. For data management, Stata version 15.0 was used.

## Results

**Figure 1.**
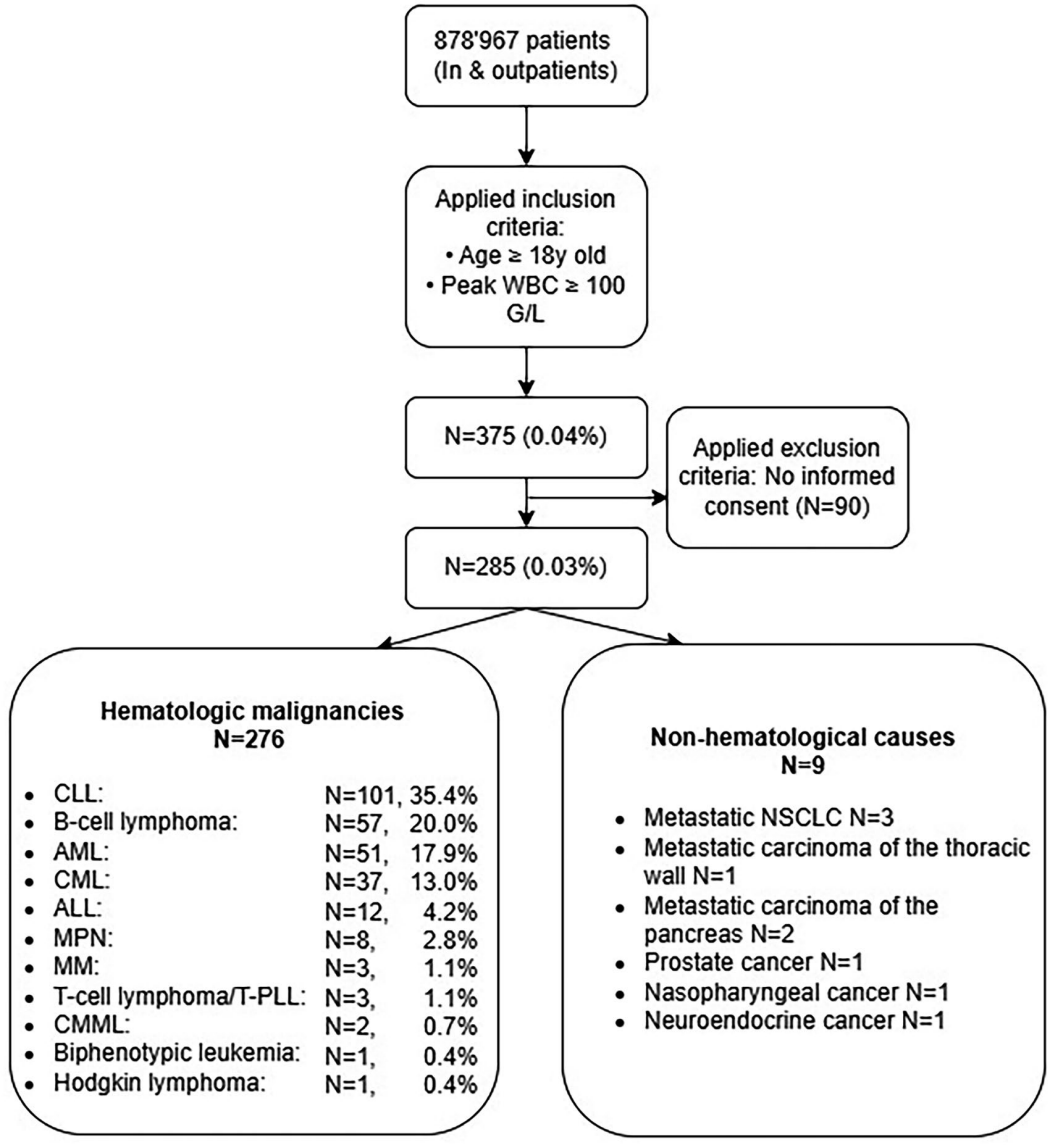
Flow-chart of included patients with HL. **Abbreviations:** ALL: Acute Lymphocytic Leukemia; AML: Acute Myeloid Leukemia; CLL: Chronic Lymphocytic Leukemia; CML: Chronic Myeloid Leukemia; CMML: Chronic Myelomonocytic Leukemia; MM: Multiple Myeloma; MPN-MDS: Myeloproliferative Neoplasms-Myelodysplastic Syndrome; NSCLC: Non-Small Cell Lung Cancer; WBC: white blood cell counts; T-PLL: T-Prolymphocytic Leukemia

After querying the files of 878,967 adult patients, we identified 375 (0.04%) patients with HL. Ninety patients meeting HL criteria were excluded due to lack of documented general consent; these patients were not included in any analyses to ensure compliance with ethical requirements. 285 did not object to general consent and comprised the population evaluated in this study (Figure 1). The median age of all HL patients was 66.8 years (range, 18.4–93.6), and 32% were female. The median WBC count was 103.3 G/L (range, 100–161). Among the 285 HL patients, 276 (97%) had an underlying hematologic disease (Figure 1). The four most frequent hematologic causes make up more than 80% of cases: Chronic Lymphocytic Leukemia, (CLL) (*N* = 101, 35.4%), other B-cell lymphomas (*N* = 57, 20.0%), acute myeloid leukemia (AML) (*N* = 51, 17.9%) and chronic myeloid leukemia (CML), (*N* = 37, 13.0%) (Figure 1). Nine patients had a non-hematologic underlying cause, their median age was 64 years (range, 48–71, Sex: male 7 (78%), female 2 (22%)), and the majority were male (78%). All nine patients demonstrated neutrophil predominance. All nine patients had an underlying malignancy, which included non-small cell lung cancer (*n* = 3), pancreatic carcinoma (*n* = 2), thoracic wall carcinoma (*n* = 1), prostate carcinoma (*n* = 1), nasopharyngeal carcinoma (*n* = 1), and a neuroendocrine tumor (*n* = 1). Metastatic or disseminated disease was present in all cases, and 44% exhibited tumor involvement of the lung. Potential precipitants of hyperleukocytosis were primarily paraneoplastic in nature (*n* = 9), with additional cases associated with granulocyte-colony stimulating factor (G- CSF) administration (*n* = 2) and sepsis or systemic inflammatory response syndrome (*n* = 4). LDH values were available for 8 out of 9 patients; all had levels above the reference values, with 3 patients exhibiting levels over 1000 U/L. All patients had clearly elevated CRP levels. Supportive care was uniformly initiated; however, all patients succumbed to their illness within a short period after the onset of HL (1 to 24 days). The characteristics of the nine identified cases are detailed in Table 1.

**Table 1.** Laboratory and further characteristics of non-hematologic hyperleukocytosis cases.

Case	Age	Underlying disease	Trigger for HL	Peak WBC (G/L)	Hb (g/L)	Platelets (G/L)	ANC at peak HL (G/L)	Blasts	Smear findings	LDH (U/L)*	CRP (mg/L)*	Preexisting chronic leukocytosis	Early mortality	Time from peak WBC to death (days)
#1	50–55	Pancreatic cancer	Suspected infection	161	119	241	149	No	Data not available	492	90	Not tested	Yes	4
#2	65–70	Pancreatic cancer	Surgery	103	105	768	99.2	No	Anisocytosis, poikilocytosis, ovalocytes, target cells, Howell–Jolly bodies, toxic granulation, and cytoplasmic vacuolization (+)	1104	116	Yes	Yes	8
#3	65–70	NSCLC	None identified	100	86	452	Not available on the day of peak HL, 5 days before: 55% Neutrophils & 20% Eosinophils	No (data not available)	Data not available	740	213	Yes	Yes	4
#4	60–65	NSCLC	None identified	101	94	492	98.9	No	Anisocytosis,, polychromasia, poikilocytosis, toxic granulation (+)	848	304 (5 days before)	Yes	Yes	17
#5	55–60	NSCLC	Suspected infection	146	83	34	127.1	No	Anisocytosis,, poikilocytosis, toxic granulation, bas. stippling (+)	4045	273	Yes	Yes	1
#6	55–60	Thoracic malignancy	None identified	102	68	586	97.5	No	Toxic granulation (+)	411	340	Yes	Yes	24
#7	80–85	Prostate cancer	Intestinal perforation	115	92	345	89.3	0.5%	Anisocytosis,, polychromasia, poikilocytosis, toxic granulation (+)	Not performed	326	Not tested	Yes	1
#8	50–55	Nasopharyngeal cancer	G-CSF	114	117	97	96.3	0.5%	Anisocytosis,, poikilocytosis,, bas. Stippling, vacuoles (+),toxic granulation (++)	1178	130	No	Yes	2
#9	65–70	Neuroendocrine cancer	G-CSF	100	103	459	99	No	Anisocytosis, macrocytes, poikilocytosis (+)	677*	132 *	No	Yes	8

*For Case #9, laboratory values represent those available closest to the date of maximum HL. **Abbreviations:** NSCLC: non-small-cell lung cancer; G-CSF: granulocyte colony-stimulating factor; WBC: white blood cells; bas. stippling: basophilic stippling.

## Discussion

Identifying the underlying cause of HL is a key focus in consultative hematology. Here, we performed a retrospective collection of all patients identified with HL covering a period of 10 years in a tertiary university hospital. Our findings showed, as expected, that causes of HL are mainly hematologic. However, non-hematologic causes, although unusual, exist and should not be overlooked. All identified non-hematologic cases were associated with an underlying non hematologic neoplasm, mainly carcinomas in an advanced disease stage. However, a number of accompanying factors that may have contributed to the development of HL, such as infection, acute clinical complications, surgical interventions (especially abdominal), previous splenectomy, and administration of G-CSF were observed and in some of the included patients, leukocytosis was preexisting. In some cases, more than one factor coexisted, complicating causal attribution. HL cases were mainly characterized by marked neutrophilia, sometimes accompanied by mild monocytosis; relevant eosinophilia was observed in only one case. Importantly, none of the patients classified as having non-hematologic HL underwent bone marrow examination. This means that occult hematologic neoplasia, therapy-related myeloid disease, or early myeloproliferative neoplasms cannot be definitively excluded. However, the rapid onset of HL with neutrophilia did not suggest a hematologic disease per se. In addition, this is a retrospective study in which we report the data found in each patient’s files. The occurrence of HL in severely ill patients with advanced cancer and poor prognosis may explain the limited work-up to clarify the underlying causes. In two of the cases presented in this manuscript, patients received G-CSF, a factor that likely contributed to the development of HL in this setting.

Our study is the first investigating the causes of HL systematically in all patients seen at a referral hospital. A study analyzing the prevalence and causes of WBC > 35 G/L in a 400-bed hospital from 2015 to 2021 found that only 80 patients had WBC > 35 G/L; the number of screened patients was not described in the study. Furthermore, they reported that only 17/80 (20%) patients had WBC > 50 G/L, of which 71% had malignant diseases, 23% had infections, and 6% had other causes [4]. Importantly, these leukocyte thresholds are substantially lower than the ≥100 G/L criterion used in the present study, limiting direct extrapolation to true HL as defined here. This discrepancy should be considered when comparing etiologic distributions. Nonetheless, very few case reports have also demonstrated the presence of non-hematologic HL causes. Non-hematologic causes of HL reported in these anecdotal cases included severe infection mimicking leukemia, adenovirus-associated pertussis-like syndrome, paraneoplastic leukocytosis in a melanoma patient receiving immunotherapy, and hypereosinophilic pneumonitis with acute respiratory distress syndrome, respectively [9–13]. Although cases of HL in patients with isolated severe infections have been reported as described above [9,10], we found no such cases in our cohort (Table 1). The absence of isolated infection-driven HL in our cohort likely reflects the stringent WBC threshold applied (≥100 G/L). In fact, many cases reported in the literature as HL had WBC below the cutoff used in our study [4]. Furthermore, our institution, as a tertiary hospital, is characterized by an interdisciplinary approach to patient management with rapid, systematized strategies, which may have contributed to our findings.

The presentation of acute HL in certain hematologic conditions may represent a medical emergency associated with high morbidity and mortality, requiring urgent management including the use of leukapheresis [3,14]. Accordingly, HL should prompt immediate peripheral blood smear review and early hematology consultation. In our cohort, HL appeared as a terminal event in severely ill cancer patients, none of whom showed definitive HL-related symptoms. This aligns with a predominance of neutrophils, which are not typically associated with leukostasis. Paraneoplastic HL is driven by the overproduction of G-CSF and cytokines like interleukin-6 [15]. A plausible biological mechanism is that the production of G-CSF leads to sustained autocrine stimulation and myeloid proliferation, which may most likely explain leukocytosis in patients with disseminated cancer. Cytokine overproduction may explain the HL in the patients of our cohort, given the advanced stage of the disease and the poor outcomes observed. However, in the absence of direct cytokine measurements these mechanisms should be regarded as biologically plausible hypotheses rather than confirmed drivers in the present cohort. In a large retrospective study involving 758 solid tumor patients with leukocytosis (> 40 G/L), 77 (10%) were found to have a paraneoplastic HL, often presenting with neutrophil predominance and metastatic disease. These patients had a poor prognosis, with 78% dying or entering hospice within 12 weeks unless they received effective antineoplastic therapy [5]. Again, the lower leukocyte threshold (>40 G/L) limits direct comparison with our ≥100 G/L cohort.

Our study has several limitations, mainly related to its retrospective nature, also due to the reliance on electronic health records, some underlying causes of HL may have been underdiagnosed or misclassified. The absence of bone marrow evaluation in non-hematological HL cases as well as limited work-up is some cases represent limitations from a diagnostic validity perspective. However, the rapid onset of HL with neutrophilia in most of the cases did not suggest a hematologic disease per se. The rarity of non-hematological HL limits the ability to perform comparative statistical analysis. Furthermore, the diagnostic procedure, as well as the decision to treat each patient, was at the discretion of the treating physician. Consent-related exclusion may bias estimates of etiologic distribution and short-term outcomes. Although misclassification due to missing or incorrect ICD-10 codes cannot be entirely excluded, we consider this risk to be minimal. In Switzerland, hospital reimbursement is directly linked to accurate ICD-10 coding, which provides a strong incentive for complete and correct diagnostic coding. The strength of this study lies in the large number of patients screened and the access to all laboratory values, imaging results, and clinical information from the medical records, avoiding underreporting.

In conclusion, HL should immediately prompt urgent evaluation for hematologic malignancy, with immediate peripheral blood smear review and early hematology consultation. HL exceeding 100 G/L, as defined in our study, is uncommon in non-hematologic conditions, but can occur in advanced solid malignancies, particularly in terminal stages with inflammatory reactivity or following G-CSF treatment. Isolated infection-related HL was not observed in our cohort, likely reflecting the stringent threshold (≥100 G/L) and tertiary-center setting.

## Institutional review board statement

In accordance with Swiss regulations, only patients who had provided general consent for the retrospective use of their health-related data for research purposes, or who had not formally opted out, were included in the study. The study received ethical approval from the Ethics Committee in Bern and the requirement for study-specific written informed consent was waived by the competent ethics committee.

## Data Availability

For this study, there are no publicly archived datasets analysed or generated during the study. We will make the data collected during this research available upon reasonable request.
